# Isolation, molecular profiling, and antimicrobial sensitivity patterns of ESBL producing *Acinetobacter baumannii* in wastewater discharges from Goranchatbari sub-catchment area in Dhaka

**DOI:** 10.1371/journal.pone.0341652

**Published:** 2026-02-06

**Authors:** Amanta Rahman, Nayeema Haque, Mohammed Tanveer Hussain, Md. Sakib Hossain, Ahmed Ishtiaque Amin Chowdhury, Md. Foysal Abedin, Tahani Tabassum, Md. Hajbiur Rahman, Mohammad Atique Ul Alam, Mohammad Rafiqul Islam, Iftekhar Bin Naser, Md. Shafiqul Islam, Zahid Hayat Mahmud

**Affiliations:** 1 Laboratory of Environmental Health, Health System and Population Studies Division, International Centre for Diarrheal Disease Research, Bangladesh (icddr, b), Dhaka, Bangladesh; 2 Biotechnology Program, Department of Mathematics and Natural Sciences, BRAC University, Mohakhali, Dhaka, Bangladesh; 3 Department of Biological Sciences, Louisiana State University, Life Sciences Building, Baton Rouge, Louisiana, United States of America; 4 Department of Pathobiological Sciences, School of Veterinary Medicine, Louisiana State University, Baton Rouge, Louisiana, United States of America; 5 Institute of Water and Flood Management (IWFM), Bangladesh University of Engineering and Technology (BUET), Dhaka, Bangladesh; University of Chittagong, BANGLADESH

## Abstract

The World Health Organization has recommended the development of new antimicrobials against *Acinetobacter baumannii*, one of the six highly significant pathogens. In this study, 28 wastewater samples were collected from the Goranchatbari sub-catchment area in Dhaka city over several periods. The samples were characterized for the presence of *Acinetobacter* spp. The isolates showing cream, opaque colonies on CHROMagar^TM^ ESBL plates were considered positive for ESBL production. They were further characterized for major antibiotic resistance and pathogenic genes, biofilm production and antibiotic susceptibility testing. Furthermore, the isolates were phylogenetically clustered based on their Enterobacterial Repetitive Intergenic Consensus (ERIC) profiles and correlation matrix analysis was performed. Out of 28 samples, 27 were positive for *Acinetobacter* spp. and a total of 106/249 (42.6%) sample representative isolates were positive for ESBL production. Out of these 106 isolates, 97 (91.5%) were genotypically confirmed to belong to the *Acinetobacter* spp*.* and of which, 72 (74.2%) were genotypically confirmed as *Acinetobacter baumannii*. Among the distribution of β-lactamase genes, *bla*_TEM_ was the most prevalent being present in 40/72 (55.6%) isolates, followed by *bla*_SHV_ in 3/72 (4.2%) isolates. With respect to the pathogenic genes, *pgaB* and *bfmS* were the most prevalent being present in 80.6% and 69.4% of the isolates, respectively. The antibiotic susceptibility testing revealed a diverse range of resistance patterns with high levels of intermediate resistance being observed for cefotaxime and ceftriaxone. The biofilm formation screening revealed that 48.6% and 44.4% of isolates formed strong biofilms at 37°C and 25°C, respectively. The phylogenetic clustering of the *A. baumannii* isolates resulted in the formation of 10 clusters at 60% similarity index, and the correlation matrix helped reveal important associations between genotypic and phenotypic traits. These results demonstrate the continued prevalence of *A. baumannii* within these environmental reservoirs and its ability to persist despite seasonal variations, prioritizing changes in environmental health policies that aim to reduce the widespread prevalence of these pathogens.

## 1. Introduction

In recent years, the rise of antimicrobial resistance (AMR) in bacteria has become a critical health concern around the world, with at least 1.27 million direct reported deaths worldwide [1]. This figure likely underestimates the true impact due to inadequate reporting and surveillance with the situation worsening after the COVID-19 pandemic [2]. Researchers predicted that without effective intervention, by 2050, over 10 million deaths per year would result directly from AMR globally [3]. The widespread prevalence of antibiotic resistance not only threatens the stability of healthcare systems but also could make treating infections increasingly difficult, leading to significant societal and economic consequences [4].

The genus *Acinetobacter* is a family of gram-negative bacteria, characterized by its strictly aerobic, non-motile coccobacilli morphology [4]. It thrives on simple microbiological media and is frequently found in diverse surroundings, which include soil and surface water [5,6]. However, not all species of *Acinetobacter* naturally reside in environmental habitats, as *Acinetobacter baumannii* is not generally found in nature and is mostly isolated from infected individuals and hospital settings [7].. Due to the organism’s ability to colonize environmental surfaces in healthcare facilities and persistence for prolonged periods, it is a significant concern in nosocomial settings and healthcare-associated infections [8].

The prevalence of *Acinetobacter* spp. in healthcare settings is well documented but their AMR patterns in natural settings remain poorly understood. This is of particular concern since the World Health Organization (WHO) classified *A. baumannii* as the highest priority in its overall priority list of antibiotic-resistant bacteria in 2017 [9]. Evidence suggests that *Acinetobacter* strains can spread resistance traits among surrounding populations [10], hence, the detection of *A. baumannii* has increased drastically from environments. Hospital effluent is determined to be the primary source of *A. baumannii* with clinical significance where confirmed presence of carbapenem-resistant *Acinetobacter* spp. was also observed [10–12]. Nevertheless, despite the frequent exposure of hospital waste into the water bodies and their close proximity, there has been limited investigation to detect the presence of similar resistant isolates from water bodies.

*A. baumannii* has been categorized among the ESKAPE group *(Enterococcus faecium*, *Staphylococcus aureus*, *Klebsiella pneumoniae*, *A. baumannii*, *Pseudomonas aeruginosa* and *Enterobacter* spp.), known for developing exceptional ability in acquiring resistance against drugs through different mechanisms, such as production of β-lactamases, efflux pumps, and biofilm formation, which develops increased resistance towards antimicrobials, posing a significant threat to human health [13]. Conventionally, *A. baumannii* infections are usually managed by the use of carbapenems, aminoglycosides, fluoroquinolones, and in the case of MDR, by the use of polymyxins [14]. One important mechanism to develop antimicrobial resistance is by employing enzymes like extended spectrum β-lactamases (ESBLs) [15]. ESBLs, falling under class-A β-lactamases, can hydrolyze third generation cephalosporins and other β-lactams [16]. There are three primary ESBL enzymes—CTX-M, SHV, and TEM, which are more commonly characterized by resistant bacteria isolated from environmental samples [17]. Moreover, OXA-type β-lactamases are prevalent in the genomes of *A. baumannii* and make contributions, especially in the resistance against oxacillin and carbapenem [18]. Several recent studies have reported a higher prevalence of ESBL genes in *Acinetobacter* species isolated from both environmental and clinical settings [10,19]. This increasing prevalence of ESBL-producing *A.* *baumannii* strains raises significant concerns, especially in developing countries like Bangladesh considering the limited healthcare facilities and future implications that such difficult infections might have.

Despite the extensive research on the epidemiology and resistance pattern of *A. baumannii* strains, there is a limited understanding regarding its pathogenicity traits and virulence patterns [20]. Various mechanisms have been proposed to emphasize its role in colonization, infection, and epidemic spread. However, biofilm formation on both different biotic and abiotic surfaces has been a major concern, leading to chronic and persistent infections as well as AMR [21]. Studies suggested the significance of several virulence factors correlating with *A. baumannii* biofilm formation that include outer membrane protein A (*ompA*), the two-component system (BfmS/BfmR), chaperon-usher pilus (Csu), poly-β-(1,6)-N-acetyl glucosamine (PNAG), biofilm-associated protein (Bap), extracellular exopolysaccharide (EPS), and quorum-sensing systems [22,23]. Bap is a large surface protein involved in cell-to-cell interaction and biofilm maturation, *ompA* encodes the major porin of *A. baumannii* which plays roles in biofilm formation and persistence, serum resistance, induction of apoptosis, and antimicrobial resistance. It also plays a crucial role in attachment and invasion of epithelial cells through interaction with fibronectin. Moreover, *csuE* is associated with pilus production and biofilm formation which promotes biofilm formation on abiotic surfaces; *bla*_PER-1_ induces biofilm formation and attachment of *A. baumannii* to epithelial cells of the respiratory tract and *bfmS* is a histidine sensor kinase gene that senses environmental conditions [21]. The virulence genes investigated in this study were selected based on their frequent reporting in previous studies and their established roles in *A. baumannii* pathogenicity, biofilm formation, and environmental persistence. Genes such as *ompA*, *bap*, *bfmS*, *csuE*, and *pgaB* are well recognized for their involvement in adhesion, surface colonization, and biofilm development, which are critical for survival in both clinical and environmental settings. The remaining genes have also been commonly screened in earlier investigations [24]. Other studies also have established a positive correlation between antimicrobial resistance and formation of biofilm in *A. baumannii* [25,26]. Since biofilm formation is attributed to reduced drug penetration and an additional polymeric barrier [27,28], antimicrobial resistance within biofilms increases substantially [29]. Consequently, the heightened resistance challenges treating persistent biofilm-causing infections, even with multiple antibiotics [30].

Countries with lower socio-economic structures often encompass traits such as lesser hygiene, low awareness and poor waste management infrastructure that often exacerbates severity of AMR infections, as has been reported previously [10,19,31,32]. Considering the implications of AMR organisms in the environment of developing countries and the lack of such investigations in Bangladesh, this study aimed to detect ESBL-producing *A. baumannii* from the environment, assess their virulence genes, antibiotic susceptibility profiles, and biofilm formation capability. Additionally, molecular typing was conducted based on ERIC-PCR to characterize the genetic similarity among the virulent environmental *A. baumannii* isolates.

## 2. Materials and methods

### 2.1. Sampling sites and sample collection

**Fig 1:**
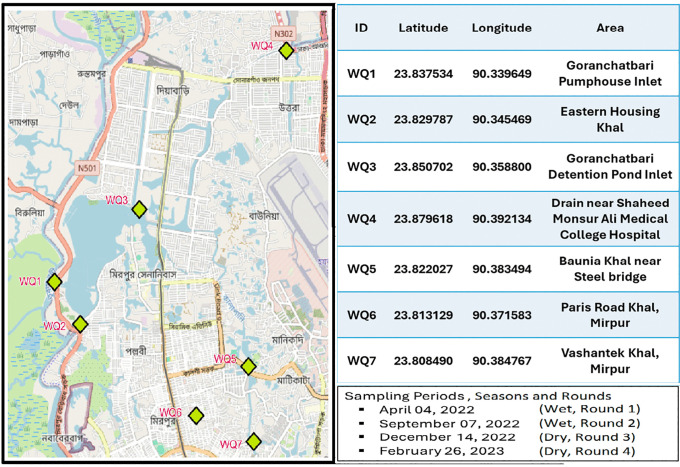
Sampling point locations in the Goranchatbari sub-catchment. Base map obtained from the USGS National Map Viewer (public domain) [Available from: https://apps.nationalmap.gov/viewer/]; sampling locations and sub-catchment boundary were added by the authors. The sampling point descriptions and sampling period information were adapted from our previous study [33], which was conducted as part of the same sampling campaign and is licensed under CC BY 4.0.

Samples collection was performed from 28 points in seven (WQ1-WQ7) distinct severed waterbodies from Goranchatbari sub-catchment area in western Dhaka city, which included canals, local drainage system and retention ponds or wetlands (Fig 1). The Goranchatbari sub-catchment was selected as the study site because it is one of the largest and most environmentally significant drainage catchments in western Dhaka. The western part of Dhaka comprises ten major sub-catchments—Goranchatbari, Rampura, Kallyanpur, Dholaikhal, Kamalapur, Nawabgonj, Shahidnagar, Sadarghat, Kamalbagh, and Basabo [34]. In Goranchatbari, stormwater and wastewater from the entire sub-catchment accumulate in a large retention pond, from which water is pumped into the adjacent Turag River [35]. As a result, the water quality of this retention pond directly influences downstream river water quality. The sampling locations were publicly accessible urban water bodies within the Goranchatbari sub-catchment area of Dhaka. These sites are not protected, privately owned, or subject to restricted access. Therefore, no specific permits were required to conduct sampling activities.

Sampling was conducted in four discrete rounds: April 2022 and September 2022 (wet season), and December 2022 and February 2023 (dry season), following the climatic seasonality of Bangladesh. A total of 28 samples were collected across these four rounds (seven samples per round). Samples were brought to the Laboratory of Environmental Health, icddr,b, Dhaka in sterile plastic bottles (NALGENE, NY, USA) while maintaining a cold chain ensuring standard procedures [33,36].

### 2.2. Sample processing

Samples processing was performed within 24 hours of collection after bringing them to room temperature. Using sterile normal saline (0.85% NaCl), a serial dilution (10^-1^, 10^-2^, 10^-3^) of samples was prepared, following which 100 μL of serial decimal dilutions was spread on CHROMagar™ Acinetobacter (CAB) agar (CHROMagar, Paris, France) plates and incubated at 37 ± 0.5°C between 18–24 hrs.

### 2.3. Isolation of ESBL-producing *Acinetobacter* spp.

After adequate incubation, colonies that gave a unique red color were presumed to be *Acinetobacter* spp*.* Using patch inoculation techniques, a maximum of 10 single, random red colonies were further transferred onto CHROMagar™ ESBL (CHROMagar, Paris, France) and CAB plates, followed by incubation at 37 ± 0.5°C between 18–24 hrs [32]. Colonies that displayed red color on CAB plates and opaque cream color on CHROMagar^TM^ ESBL plates simultaneously were chosen for further analysis and enriched overnight in Luria Bertani broth. The isolates were also stored at −70°C using 30% pre-sterilized glycerol-broth mixture for future analysis.

### 2.4. Genotypic confirmation of *Acinetobacter* spp. and *A. baumannii*

Genomic DNA was extracted from pre-enriched cultures following the boiling lysis protocol adapted from a previous study [37]. Each batch of DNA extraction contained an extraction blank. The presence of *recA* and 16S*-23S-rRNA ITS* genes were analyzed with PCR following previously published protocols [38]. The PCR techniques utilized two sets of primers; the first set comprised P-rA1 and P-rA2, targeting the conserved region of the *recA* gene for *Acinetobacter* spp. detection, while the second set comprised P-Ab-ITSF and P-Ab-ITSB targeting the 16S-23S ribosomal intergenic spacer region of *A. baumannii* [39]*.* The primer sequences are listed in S1 Table. A multiplex PCR was performed with a total reaction mixture volume of 50 μL containing 0.4 μM of each primer. The PCR conditions comprised of initial denaturation for 5 mins at 94°C, subsequently 30 cycles of denaturation for 30 secs at 95°C, annealing for 30 secs at 54°C and extension for 30 secs at 72°C followed by 7 mins elongated extension at 72°C. *A. baumannii* NCTC 12156 and no-template control (NTC) were used as positive and negative controls, respectively. The PCR reaction was conducted in a BIORAD T100™ Thermal cycler (BIORAD, CA, USA). For confirmation of PCR amplification, agarose gel electrophoresis was conducted with 1% gel and visualized on GelDoc Go Imaging System (BIORAD, CA USA) for all the PCR reactions conducted in this study. The gel image was saved for further analysis.

### 2.5. Molecular detection of antibiotic resistance genes

All 72 ESBL-producing *A. baumannii* isolates were assessed for the detection of *bla*_SHV_, *bla*_TEM_, *bla*_CTX-M_, *bla*_OXA,_ using established protocols [40]. A multiplex PCR reaction was prepared with a total volume of 50 μL containing 0.4 μM of each primer. The PCR conditions comprised 95°C for 5 mins, followed by 30 cycles of 94°C for 30 secs, 62°C for 90 secs, and 72°C for 1 min, with final extension at 72°C for 7 mins. Gene specific validated positive controls for these reactions were sourced from an earlier study [17] and NTC was used as negative control. S1 Table contained the details regarding primer sequences and their product sizes. The PCR amplification products were analysed by electroporation on Tris-Borate-EDTA (TBE) buffered agarose 1% w/v gels.

### 2.6. Molecular detection of biofilm-associated virulence genes

All 72 ESBL-producing *A. baumannii* isolates underwent molecular testing to detect the presence of ten biofilm-associated virulence genes, namely *espA*, *ompA*, *csuE*, *bfmS*, *bap*, *fimH*, *kpsMII*, *bla*_PER-1_, *ptk* and *pgaB.* Three separate multiplex PCR reactions were carried out with a total volume of 50 μL and 0.4 μM of each primer as per previously defined protocol [21]. S1 Table contains the primer sequences and their resulting product sizes. The first multiplex PCR reaction was run for *espA, bfmS, fimH* and *csuE* genes, the second multiplex PCR was run for *bla*_PER-1_*, bap, ptk* and *pgaB* genes and the third multiplex PCR was for the *ompA* and *kpsMII* genes. The PCR conditions for the 3 reaction mixtures were similar except for the annealing temperatures. The conditions were set for an initial denaturation for 5 mins at 94°C, subsequently 30 cycles of denaturation for 30 secs at 95°C, annealing for 1 min and extension for 30 secs at 72 °C followed by 7 mins elongated extension at 72°C. The annealing temperatures for the first, second and the third reaction were 60°C, 52°C and 58°C, respectively. *A. baumannii* NCTC 12156 was used as a positive control, along with NTC as negative control. The presence and subsequent amplification of the targeted genes were determined by resolving the PCR products in 1% agarose gel buffered with TBE.

### 2.7. Phenotypic detection of antimicrobial resistance profiles

Antibiotic susceptibility of 72 ESBL-producing *A. baumannii* isolates were determined following the Clinical and Laboratory Standards Institute (CLSI) guidelines using the standard Kirby-Bauer disk diffusion method [41]. Antibiotic disks (Thermo Scientific™ Oxoid™, Basingstoke, Hampshire, UK) used in the study were commercially available and employed for assessing the susceptibility patterns to 11 antimicrobial agents, imipenem (IMP, 10 μg), meropenem (MEM, 10 μg), cefepime (FEP, 30 μg), cefotaxime (CTX, 30 μg), ceftriaxone (CRO, 30 μg), ciprofloxacin (CIP, 5 μg), gentamicin (CN, 10 μg), amikacin (AK, 30 μg), tetracycline (TE, 30 μg), cotrimoxazole (SXT, 25 μg), and piperacillin/tazobactam (TPZ, 110 μg). An inoculum suspension of 1.5x10^8^ CFU/mL cell density was prepared which was standardize against 0.5 McFarland solution. The inoculum was streaked on Mueller-Hinton agar medium (Difco, MD, USA). Antibiotic disks were placed on the streaked plates and incubated at 37°C for 18 (±2 hrs). The experiment was repeated thrice with *E. coli* ATCC 25922 strain for QC purpose. The isolates were classified into sensitive, intermediate, and resistant using the mean diameter of the zone of inhibition (mm) [41].

### 2.8. Biofilm formation assay

Quantitative adherence assay was used for the biofilm formation experiment at both 25°C and 37°C for 48 hours [42]. Fresh inoculum culture was prepared for the 72 *A. baumannii* isolates by inoculating single colonies in LB broth, and incubating overnight at 37°C. Following incubation, the overnight culture was suspended in fresh LB in a 96-well microtiter plate (Costar, USA) and incubated. Uninoculated LB served as a negative control. The optical density (OD) was measured under 590 nm wavelength using an ELISA plate reader (BioTek, Vermont, USA). Following Nirwati et al., biofilm formation was categorized into either strong, moderate, weak or non-biofilm formation. The optical density cut-off value (ODc) was established from the negative values. The ODc value is three standard deviations (SD) above the mean OD of the negative control, that is ODc = average OD of negative control + 3x Standard deviation (SD) of negative controls. The isolates with OD ≤ ODc were termed non-biofilm producers. On the contrary, isolates with ODc < OD ≤ 2x ODc are categorized as weak biofilm producers, whereas 2x ODc < OD ≤ 4x ODc and OD > 4x ODc are categorized as moderate and strong biofilm producers, respectively [17].

### 2.9. ERIC-PCR genotyping of *A. baumannii*

To assess the genetic relatedness and clonal relationships among the 38 *A. baumannii* isolates carrying four or more pathogenic markers, Enterobacterial Repetitive Intergenic Consensus (ERIC)-PCR was performed using the ERIC-2 primer (5’-AAGTAAGTGACTGGGGTGAGCG-3’) following established protocols [43,44]. PCR products were then separated into several bands across a 2% agarose gel and gel image analysis was performed using GelJ v.2.0, with image normalization achieved through the Gaussian regression method. The patterns generated by the PCR products were clustered to construct a phylogenetic tree using the Dice coefficient and the unweighted pair group method using arithmetic averages (UPGMA) with 1% tolerance value.

### 2.10. Statistical analysis

Microsoft Excel (2019) and R (Version 4.5.1) were used for statistical analyses. In order to conduct the analysis, the summaries of antibiotic resistance genes and the presence or absence of both resistance and virulence genes were transformed into a binary code, where 1 represented the presence of a specific gene and 0 indicated its absence. For the correlation matrix, initially 28 variables were selected for inclusion in the correlation matrix analysis (S5 Table). For analysis, all the variables were screened for variability across the 72 isolates. As 9 variables showed identical values, that is no variation was found, so those variables were excluded from the analysis as a correlation coefficient cannot be computed with no variation. Then the remaining 19 variables were used as inputs for the correlation matrix. No data normalization steps were taken for the correlation analysis. R (version 4.5.1) was used to generate the correlation plot. For testing the Normality assumption, the data were assessed using the Shapiro-Wilk test and found that all the variables were non-normal as their p-values were less than 0.05, so Spearman’s rank correlation was applied, which is robust to non-normal data distribution. Using “readxl” package in R, the dataset was imported into R from Microsoft Excel and for visualizing the correlation matrices “corrplot” package was used in R. In R, “cor” function was used to compute the correlation matrix [45,46].

Fig 6 reveals the pairwise correlations. A pairwise Spearman correlation test was conducted using the same 19 variables considered in the correlation matrix. This was carried out using the “cor.test()” function in the R (version 4.5.1), p-values were compiled into an p-value matrix with diagonal values consisting of zeros. This matrix was exported in an Excel file and provided as the S4 Table.

**Fig 2:**
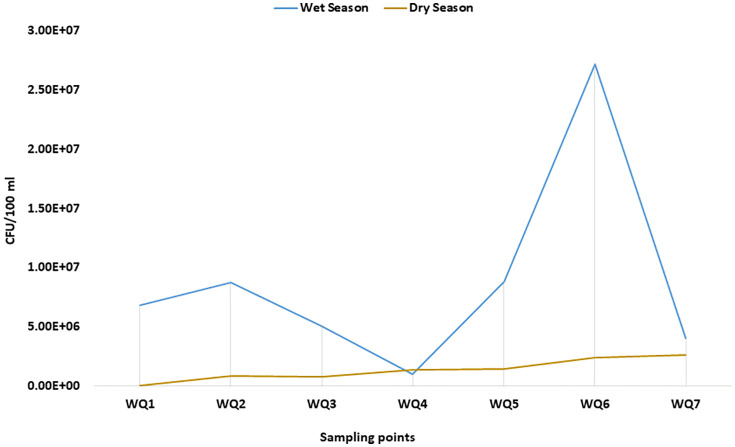
Distribution of presumptive *Acinetobacter* spp. across the seasonal variation. The prevalence of *Acinetobacter* spp. was higher during the wet season in comparison to the dry season.

**Fig 3:**
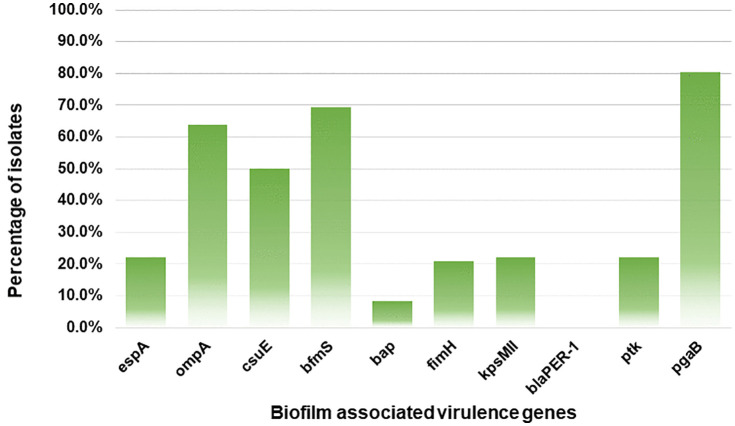
Frequency of biofilm-associated virulence genes among the isolates.

**Fig 4:**
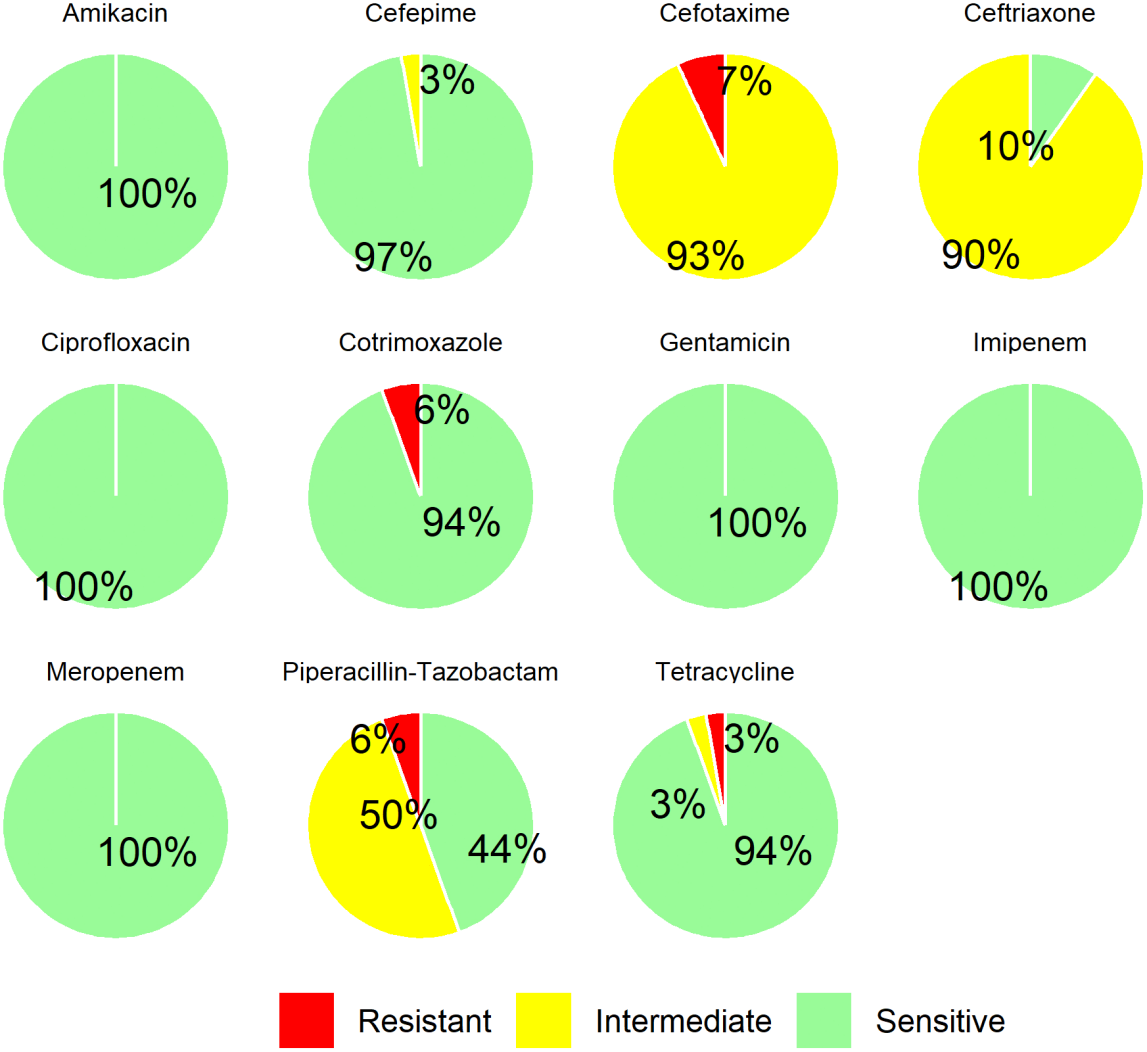
Antibiotic susceptibility pattern of *A. baumannii* isolates.

**Fig 5:**
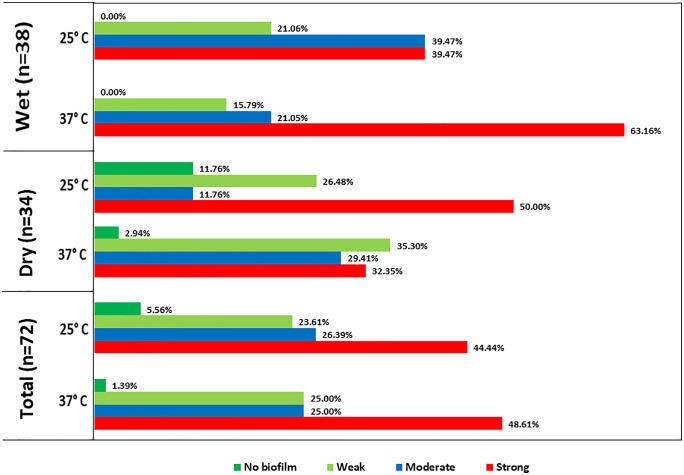
Biofilm formation capability of the isolated *A. baumannii* at 37°C and 25°C.

## 3. Results

### 3.1. Presumptive ESBL *Acinetobacter* spp. in the environmental samples

Out of the 28 samples, 27 samples indicated the presence of presumptive *Acinetobacter* spp. The organism was more abundant in the wet season than the dry season as demonstrated through phenotypic assessment (Fig 2). A total of 249 presumptive *Acinetobacter* isolates were patched onto CHROMagar^TM^ ESBL plates, and 42.6% (106/249) isolates were phenotypically positive for ESBL production.

### 3.2. Molecular assay confirms the presence of ESBL *A. baumannii* in the samples

Among the putative ESBL *Acinetobacter* isolates, 91.5% (97/106) harbored the *recA* gene, affirming them to be *Acinetobacter* spp. Among these, 74.2% (72/97) tested positive for *A. baumannii* species based on the presence of the *ITS* gene, while the remaining 25.8% (25/97) were identified as non-*baumannii* species (S2 Table). The prevalence of confirmed ESBL *A. baumannii* during the wet and dry seasons was 74.5% (38/51) and 73.9% (34/46), respectively (p = 1.0, > 0.05).

### 3.3. *bla*_TEM_ was the most prevalent antibiotic resistance gene in ESBL-producing *A. baumannii* isolates

Molecular confirmation of β-lactamase genes was performed on the 72 *A. baumannii* isolates. Among these isolates, 55.6% (40/72) tested positive for *bla*_TEM_ and 4.2% (3/72) tested positive for *bla*_SHV_ (S3 Table). The isolates were found to be negative for *bla*_CTX-M_ or *bla*_OXA_. Moreover, *bla*_TEM_ and *bla*_SHV_ were found to coexist in 2.8% (2/72) of the isolates.

### 3.4. All ESBL *A. baumannii* isolates contained biofilm-associated virulence genes

Nine out of ten target virulence genes were identified among the ESBL isolates. The *pgaB* gene was the most prevalent, detected in 80.6% (58/72) of the isolates, followed by the *bfmS* and *ompA* harbored in 69.4% (60/72) and 63.9% (46/72) of the isolates, respectively. Additionally, the *csuE* and *bap* genes were present in 50% (36/72) and 8.3% (6/72) of the isolates, respectively. None of the isolates tested positive for *bla*_PER-1_ gene (Fig 3). Only 11.1% (8/72) of the isolates carried one pathogenic gene while the remainder harbored multiple genes ranging from two to a maximum of seven genes as shown in Table 1.

**Table 1:** Percentage of biofilm related virulence genes among the isolates.

Number of Biofilm Related Virulence Genes	Patterns of Biofilm Related Virulence Genes	Percentage (%) of *A. baumannii* (N = 72)
1 gene (n = 8)	*ompA*	2.8
	*pgaB*	2.8
	*bfmS*	5.6
2 Genes (n = 9)	*fimH-pgaB*	1.4
	*bfmS-pgaB*	6.9
	*bfmS-csuE*	2.8
	*bfmS-ompA*	1.4
3 Genes (n = 17)	*bfmS-csuE-pgaB*	9.7
	*fimH-bap-ompA*	2.8
	*espA-ptk-pgaB*	1.4
	*fimH-pgaB-ompA*	4.2
	*bfmS-pgaB-ompA*	4.2
	*espA-fimH-pgaB*	1.4
4 Genes (n = 16)	*bfmS-csuE-pgaB-ompA*	5.6
	*csuE-pgaB-ompA-kpsMII*	2.8
	*bfmS-csuE-ptk-pgaB*	1.4
	*csuE-ptk-ompA-kpsMII*	1.4
	*bfmS-pgaB-ompA-kpsMII*	1.4
	*espA-fimH-pgaB-ompA*	2.8
	*bfmS-fimH-pgaB-ompA*	2.8
	*fimH-bap-pgaB-ompA*	1.4
	*fimH-ptk-pgaB-ompA*	1.4
	*espA-bfmS-pgaB-ompA*	1.4
5 Genes (n = 15)	*bfmS-csuE-pgaB-ompA-kpsMII*	5.6
	*bfmS-csuE-ptk-pgaB-ompA*	4.2
	*espA-bfmS-csuE-ptk-pgaB*	2.8
	*espA-csuE-pgaB-ompA-kpsMII*	1.4
	*espA-csuE-bap-ompA-kpsMII*	1.4
	*bfmS-ptk-pgaB-ompA-kpsMII*	1.4
	*bfmS-fimH-bap-pgaB-ompA*	2.8
	*espA-bfmS-csuE-ptk-ompA*	1.4
6 Genes (n = 6)	*espA-bfmS-csuE-pgaB-ompA-kpsMII*	2.8
	*espA-bfmS-csuE-ptk-pgaB-ompA*	2.8
	*bfmS-csuE-ptk-pgaB-ompA-kpsMII*	1.4
	*espA-csuE-ptk-pgaB-ompA-kpsMII*	1.4
7 Genes (n = 1)	*epsA-bfmS-csuE-ptk-pgaB-ompA-kpsMII*	1.4

N = total number of isolates, n = number of isolates containing a specific number of genes.

### 3.5. Majority of the isolates exhibited higher resistance towards third generation cephalosporins

Several classes of antimicrobials were tested against the 72 *A. baumannii* isolates, revealing that all the isolates were sensitive to amikacin, imipenem, meropenem, gentamicin, and ciprofloxacin. A high proportion, 97% (70/72) exhibited susceptibility to cefepime, while 94% (68/72) were individually susceptible to cotrimoxazole and tetracycline. Notably, only 44% (32/72) were susceptible to piperacillin-tazobactam and merely 10% (7/72) showed susceptibility to ceftriaxone. None of the isolates were sensitive to cefotaxime with 7% (5/72) demonstrating strong resistance according to CLSI guidelines (Fig 4).

### 3.6. Biofilm forming capabilities of ESBL producing *A. baumannii*

The biofilm formation assay was conducted for all ESBL-producing *A. baumannii* isolates at both 25°C and 37°C. At 25°C, 44.4% (32/72) isolates exhibited strong, 26.4% (19/72) moderate, 23.6% (17/72) weak and 5.6% (4/72) no biofilm formation. Whereas at 37°C, 48.6% (37/72) isolates were categorized as strong, 25% (18/72) moderate to weak and 1.4% (1/72) no biofilm formers. Comparatively, 37°C temperature showed better biofilm forming capacity among isolates as depicted in Fig 5. Notably, all isolates obtained during the wet season were capable of forming biofilm, whereas several dry-season isolates were non-biofilm formers, indicating a comparatively higher overall biofilm-forming capacity among wet-season isolates.

### 3.7. Correlation matrix analysis

**Fig 6:**
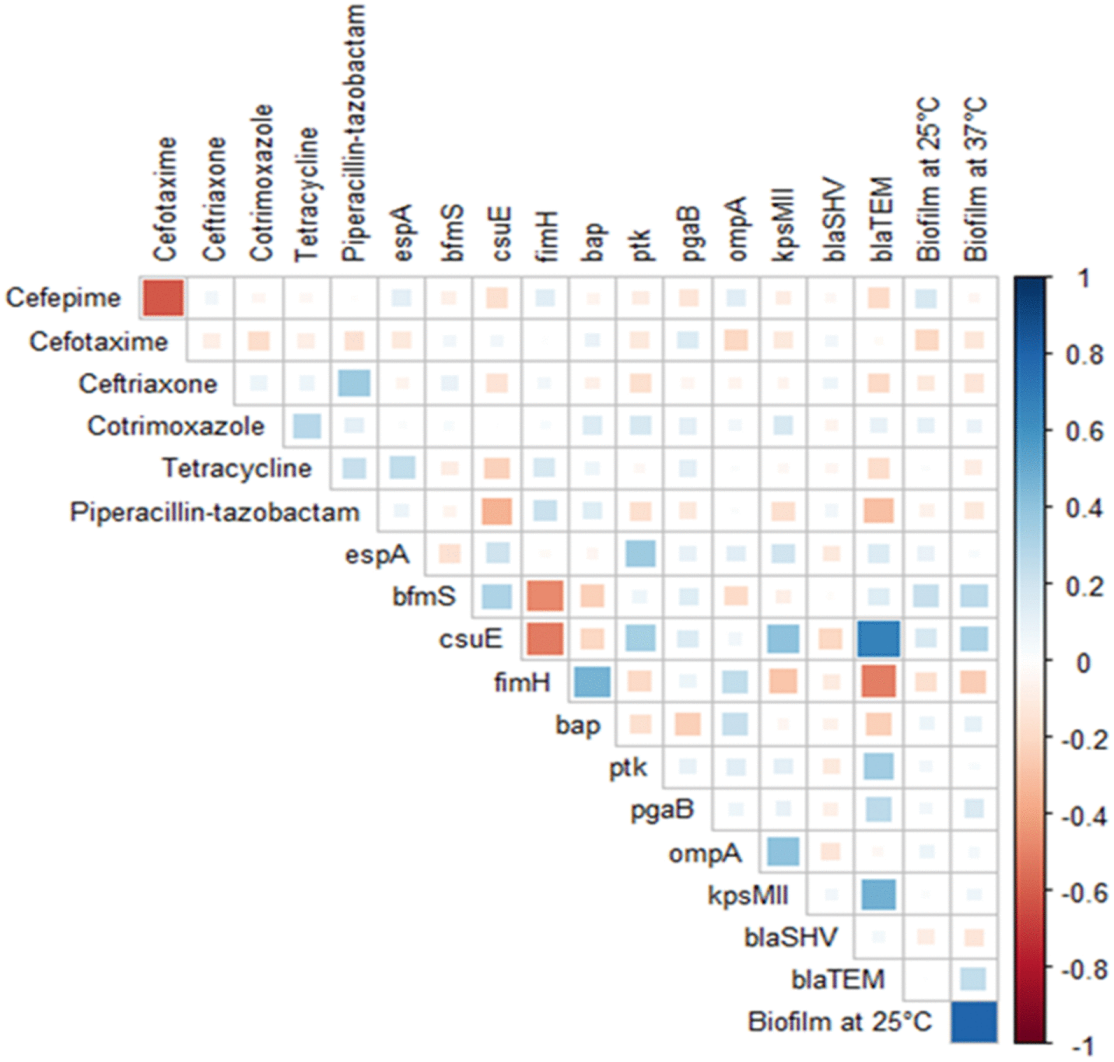
Correlation matrix of phenotypic (antibiotic resistance profiles and biofilm formation) and genotypic (resistance and virulence genes) traits of *A. baumannii* portrays the correlation among the variables. Blue squares indicate a positive correlation and red squares are indicative of a negative correlation. The size and strength of the color represent the numerical value of the correlation coefficient.

A correlation matrix was constructed to understand the relation between the phenotypic and genotypic traits. The results of the analysis revealed positive correlations between the presence of pathogenic genes and phenotypic resistance to antibiotics (Fig 6). The presence of *bla*_TEM_ gene was positively correlated with the presence of pathogenic genes such as *csuE, kpsMII, ptk,* and *pgaB*. Similarly, the formation of biofilm was positively correlated with the presence of *csuE and bfmS* genes. Likewise, if the isolates formed biofilm at 25°C, they were also likely to form biofilm at 37°C. However, the presence of *bla*_TEM_ and *bla*_SHV_ showed no significant correlation with ceftriaxone and cefotaxime. The significance of correlations between variables was shown in S4 Table.

### 3.8. Genetic relatedness of *A. baumannii* isolates

**Fig 7:**
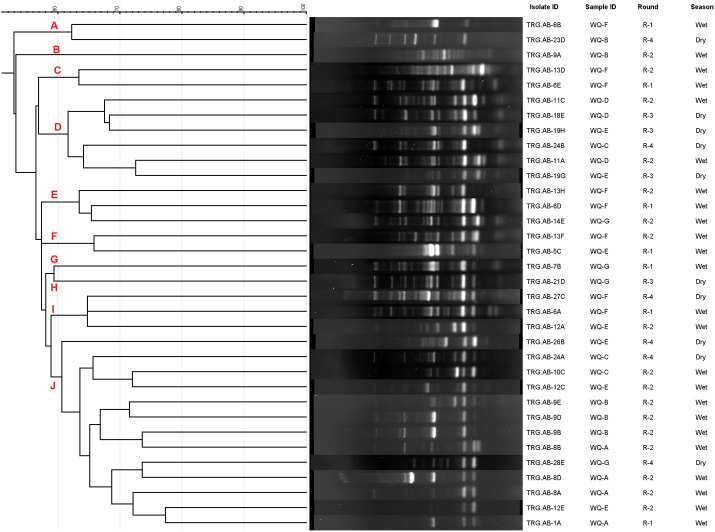
DNA fingerprinting pattern obtained using ERIC-PCR. Considering a 60% similarity index, the isolates were grouped into 10 clusters **(A-J)**.

Genetic relatedness among the *A. baumannii* isolates carrying four or more pathogenic genes was explored using ERIC-PCR. Following repetitive PCR, 4 of the isolates did not produce any bands, as a result genetic profiles of 34 virulent isolates were analyzed. Following dendrogram analysis (Fig 7), 10 different clusters were obtained where the isolates showed a 60% similarity index. The isolates produced amplicons ranging from 4–19 per isolate, and among them, 400 and 500 bp were most common. The largest cluster-J contained a total of 13 isolates. Notably, several isolates obtained from multiple rounds of sampling were grouped under the same cluster. For instance, isolates TRG.AB-6B and TRG.AB-23D were grouped under the same cluster although being isolated during different seasons of the year, suggesting that they likely extend from the same clonal lineage within the sampling points. Similarly, the isolates TRG.AB-13F and TRG.AB-5C were grouped under the same cluster. Furthermore, several *A. baumannii* isolates obtained during different sampling seasons were also grouped under the same cluster.

## 4. Discussion

In recent years, an alarming emergence of MDR [47,48] and biofilm-forming *A. baumannii* has been observed*,* which facilitates persistent infections [49]. This study, therefore, aimed at identifying the virulence factors and biofilm formation in ESBL-producing *A. baumannii* isolated from the largest sub-catchment area of Dhaka City. From the 28 environmental samples processed over the four rounds of sampling, 91.5% of the putative ESBL-producing *Acinetobacter* isolates were confirmed to belong to the *Acinetobacter* genus. Among these, 74.2% were simultaneously identified as *A. baumannii* and ESBL producer. Molecular detection also gave insight that during the wet season, the prevalence of *A. baumannii* was 74.5% compared to 73.9% during the dry season. Contrary to previous studies, stating the pathogen is more commonly prevalent in the wet season [50], this study implied that throughout the wet and dry seasons, the rate of appearance of the pathogen is almost similar.

*A. baumannii* has several mechanisms rendering antibiotics ineffective, comprising the synthesis of ESBLs, carbapenemases and metallo β-lactamases [51,52]. Within the ESBL category, the primary groups responsible for causing significant infections include *bla*_SHV_ and *bla*_TEM_, however, recent investigations indicated a rise in the prevalence of hospital-acquired infections carrying the *bla*_CTX-M_ gene [24,53,54]. In this study, *bla*_TEM_ was identified as the predominant gene present in 55.7%, followed by *bla*_SHV_ in 4.2% of the isolates. None of the isolates were found to harbor *bla*_CTX-M_ or *bla*_OXA_ genes. Although limited research has been conducted on environmental ESBL-producing *A. baumannii*, this study result aligns with previous findings of a study based on United Kingdom on clinical samples from which have shown *bla*_TEM_ to be the most prevalent β-lactamase gene in *A. baumannii*, followed by *bla*_SHV_ [55].

A number of virulence genes such as *bap*, *espA*, *bfmS*, *ompA*, and *csuE* are well-known in the biofilm formation of MDR clinical *A. baumannii* isolates [22,23,56]. PCR analysis revealed that more than 85% of the isolates carried at least two virulence genes. The *pgaB* gene was most frequently (80.6%) expressed, which highlights the necessity of the gene for poly-β-1,6-Nacetylglucosamine (PNAG) operon production and biofilm formation [57]. The *bfmS* gene was the second most predominant (69.4%), which regulates the csuA/BABCDE pilus usher-chaperon assembly system [58]; followed by the *csuE* gene (50%) involved in attachment and biofilm formation [49]. Notably, drug resistance in *A. baumannii* is associated with *ompA* gene [59], which was highly prevalent in our isolates (63.9%) even though the isolates were not MDR. None of the isolates harbored the *bla*_PER-1_ gene as reported in other studies [21,22,26,56].

All the *A. baumannii* isolates were susceptible to amikacin, ciprofloxacin, gentamicin, imipenem and meropenem. Only one isolate exhibited resistance to 3 antimicrobials (cefotaxime, cotrimoxazole, and tetracycline), thus meeting the criteria for classifications as MDR. Despite all the 72 isolates being ESBL producers phenotypically, the resistance could not be explained by the disk diffusion assay since the CHROMagar^TM^ ESBL plates detect resistance to a few third-generation antibiotics. This finding of isolates susceptible to imipenem and meropenem (both member of the carbapenem group) complies with a previous study conducted in Europe where only 2% of *Acinetobacter* spp*.* isolates showed resistance to carbapenems [24]. On the contrary, a study in South Africa reported a higher resistance against carbapenems, cephalosporins, and cotrimoxazole [60].

Biofilm formation capacity of pathogenic bacteria has been significantly associated with the severity of the infections as well as enhanced persistence of the isolates. In our study, the isolates demonstrated a comparatively higher percentage of biofilm formation at 37°C than 25°C. Previous evidence suggests that clinical *A. baumannii* isolates form stronger biofilms than environmental isolates [48], however, our study demonstrates that the environmental *A. baumannii* isolates can also form strong biofilms. In addition, the study also assessed the correlation between biofilm formation capability and antimicrobial resistance of the isolates. A negative correlation exists between isolates that were resistant to the tested antibiotics and strong biofilm-forming tendencies. The findings of this study found similarity with a previous one, where the non-MDR *A. baumannii* strains produced stronger biofilm compared to their MDR strains [25,61]. The correlation matrix revealed positive correlations between the existence of virulence determinants and antibiotic-resistance genes, which shows consistency with previous studies [62,63].

The genetic fingerprint patterns for the *A. baumannii* isolates revealed 10 clusters at a 60% similarity index, bearing different phenotypic and genotypic traits into consideration. The largest cluster-J contained a total of 13 isolates obtained during different sampling periods and points. These findings are in coherence with previous studies, which have shown relatedness between isolates obtained from distinct sampling points [64]. Furthermore, isolates obtained during different sampling seasons were grouped under the same cluster, providing evidence of environmental persistence despite seasonal fluctuations.

This research offers an extensive evaluation of ESBL-producing *A. baumannii* in an urban surface water ecosystem based on an integrated approach that employs phenotypic characterization, virulence gene profiling, and ERIC-PCR analyses for the evaluation of the genetic relatedness. Through the evaluation of the isolates during the wet and dry seasons, the study captures important temporal variations and contributes valuable insights into the persistence, health risks, and seasonal dynamics of AMR *A. baumannii*, which are little understood for low-resource, high-density urban ecosystems. There are a few limitations of this study. Firstly, this study’s findings cannot be broadly generalized considering only one municipal waste area was targeted for the analysis. A broader sampling area across Dhaka city could have augmented statistical significance. Secondly, only four major ESBL genes were selected for PCR analysis, so the presence of several other such resistance gene were not verified. Thirdly, this study did not analyze the presence of plasmids, hence, plasmid-mediated resistance was left unchecked. Moreover, a comparative analysis of the environmental isolates with the clinical isolates or hospital surrounding environments could have established an association among the spread of these organisms from one environment to another.

## 5. Conclusion

The findings in this study highlight the widespread presence of *A. baumannii* having ESBL producing capability in the environment, emphasizing the urgent need for intervention to prevent their transmission. Subsequent research should focus on monitoring the prevalence of ESBL *A. baumannii* in diverse sources from the environment. Moreover, plasmid profiling and conjugation experiment can be investigated to understand the resistance and virulence factor dissemination which will ultimately aid in reducing antimicrobial resistance.

### 5.1. Generative AI statement

The author(s) declare that no Gen AI was used in the creation of this manuscript.

## Supporting information

S1 Table List of primers for the PCR analysis conducted in the study. (DOCX)

S2 Table Distribution of ESBL-producing *Acinetobacter baumannii* isolates during the wet and dry seasons. (XLSX)

S3 Table Distribution of resistance and biofilm-associated virulence genes. (XLSX)

S4 Table The significance of correlations between variables. (XLSX)

S5 Table Variables used as inputs for correlation. (XLSX)

S6 Table Antibiotics used in this study and the interpretation of resistance. (XLSX)

S7 Table Interpretation of biofilm formation capacity of the isolates. (XLSX)

S1 Data The raw representative gel images supporting all blot and gel results reported in the article’s figures and supporting information files. (PDF)
