# Supplementary material for: Isolation, molecular profiling, and antimicrobial sensitivity patterns of ESBL producing Acinetobacter baumannii in wastewater discharges from Goranchatbari sub-catchment area in Dhaka
Source: PLoS One. 2026 Feb 6;21(2):e0341652. doi: 10.1371/journal.pone.0341652 (PMC12880640; doi:10.1371/journal.pone.0341652)
Supplement: S1 Table — (DOCX) [file pone.0341652.s001.docx]

**S1 Supplementary Table. List of primers for the PCR analysis conducted in the study.**

|  | Target Gene | Primer Name | Nucleotide Sequence (5’-3’) | Product size (bp) | Reference |
| --- | --- | --- | --- | --- | --- |
| **Species Specific Primers** | *recA* | rA1 | CCTGAATCTTCTGGTAAAAC | 425 | [1] |
|  |  | rA2 | GTTTCTGGGCTGCCAAACATTAC |  |  |
|  | *16S-23S-rRNA ITS* | Ab-ITS-F | CATTATCACGGTAATTAGTG | 208 | [1,2] |
|  |  | Ab-ITS-B | AGAGCACTGTGCACTTAAG |  |  |
| **ESBL Primers** | *bla*_SHV_ | SHV-F | CTTTATCGGCCCTCACTCAA | 237 | [3] |
|  |  | SHV-R | AGGTGCTCATCATGGGAAAG |  |  |
|  | *bla*_TEM_ | TEM-F | CGCCGCATACACTATTCTCAGAATGA | 445 | [3] |
|  |  | TEM-R | ACGCTCACCGGCTCCAGATTTAT |  |  |
|  | *bla*_CTX-M_ | CTX-M-F | ATGTGCAGYACCAGTAARGTKATGGC | 593 | [3] |
|  |  | CTX-M-R | TGGGTRAARTARGTSACCAGAAYCAGCGG |  |  |
|  | *bla*_OXA_ | OXA-F | ACACAATACATATCAACTTCGC | 813 | [3] |
|  |  | OXA-R | AGTGTGTTTAGAATGGTGATC |  |  |
| **Biofilm associated Virulence Gene Primers** | *epsA* | epsA-F | AGCAAGTGGTTATCCAATCG | 451 | [4] |
|  |  | epsA-R | ACCAGACTCACCCATTACAT |  |  |
|  | *ompA* | ompA-F | CGCTTCTGCTGGTGCTGAAT | 531 | [4] |
|  |  | ompA-R | CGTGCAGTAGCGTTAGGGTA |  |  |
|  | *bla*_PER-1_ | bla_PER-1_-F | ATGAATGTCATTATAAAAGC | 927 | [5] |
|  |  | bla_PER-1_-R | AATTTGGGCTTAGGGCAAGAAA |  |  |
|  | *bap* | bap-F | TACTTCCAATCCAATGCTAGGGAGGGTACCAATGCAG | 1225 | [6] |
|  |  | bap-R | TTATCCACTTCCAATGATCAGCAACCAAACCGCTAC |  |  |
|  | *bfmS* | bfmS-F | TTGCTCGAACTTCCAATTTATTATAC | 1428 | [6] |
|  |  | bfmS-R | TTATGCAGGTGCTTTTTTATTGGTC |  |  |
|  | *ptk* | ptk-F | GGCTGAGCATCCTGCAATGCGT | 597 | [7] |
|  |  | ptk-R | ACTTCTGGAGAAGGGCCTGCAA |  |  |
|  | *pgaB* | pgaB-F | AAGAAAATGCCTGTGCCGACCA | 490 | [7] |
|  |  | pgaB-R | GCGAGACCTGCAAAGGGCTGAT |  |  |
|  | *fimH* | fimH-F | TGCAGAACGGATAAGCCGTGG | 870 | [8] |
|  |  | fimH-R | GCAGTCACCTGCCCTCCGGTA |  |  |
|  | *kpsMII* | kpsMII-F | GCGCATTTGCTGATACTGTTG | 272 | [8] |
|  |  | kpsMII-R | CATCCAGACGATAAGCATGAGCA |  |  |
|  | *csuE* | csuE-F | ATGCATGTTCTCTGGACTGATGTTGAC | 976 | [9] |
|  |  | csuE-R | CGACTTGTACCGTGACCGTATCTTGATAAG |  |  |

**References**

1. Chen T-L, Sin L-K, Wu R-C, Shaio M-F, Huang L-Y, Fung C-P, et al. Comparison of one-tube multiplex PCR, automated ribotyping and intergenic spacer (ITS) sequencing for rapid identification of Acinetobacter baumannii. Clin Microbiol Infect. 2007;13: 801–806.

2. Tsai H-C, Chou M-Y, Shih Y-J, Huang T-Y, Yang P-Y, Chiu Y-C, et al. Distribution and genotyping of aquatic Acinetobacter baumannii strains isolated from the Puzi River and its tributaries near areas of livestock farming. Water. 2018;10: 1374.

3. Fang H, Ataker F, Hedin G, Dornbusch K. Molecular epidemiology of extended-spectrum β-lactamases among Escherichia coli isolates collected in a Swedish hospital and its associated health care facilities from 2001 to 2006. J Clin Microbiol. 2008;46: 707–712. doi:10.1128/JCM.01943-07

4. Toledo-Arana A, Valle J, Solano C, Arrizubieta MJ, Cucarella C, Lamata M, et al. The enterococcal surface protein, Esp, is involved in Enterococcus faecalis biofilm formation. Appl Environ Microbiol. 2001;67: 4538–4545.

5. Tayabali AF, Nguyen KC, Shwed PS, Crosthwait J, Coleman G, Seligy VL. Comparison of the virulence potential of Acinetobacter strains from clinical and environmental sources. PLoS One. 2012;7: e37024.

6. Lee H-W, Koh YM, Kim J, Lee J-C, Lee Y-C, Seol S-Y, et al. Capacity of multidrug-resistant clinical isolates of Acinetobacter baumannii to form biofilm and adhere to epithelial cell surfaces. Clin Microbiol Infect. 2008;14: 49–54.

7. Liou M-L, Soo P-C, Ling S-R, Kuo H-Y, Tang CY, Chang K-C. The sensor kinase BfmS mediates virulence in Acinetobacter baumannii. J Microbiol Immunol Infect. 2014;47: 275–281.

8. Bahador A, Bazargani A, Taheri M, Hashemizadeh Z, Khaledi A, Rostami H, et al. Clonal lineages and virulence factors among Acinetobacter baumannii isolated from Southwest of Iran. J Pure Appl Micribiol. 2013;7: 1559–1566.

9. Braun G, Vidotto MC. Evaluation of adherence, hemagglutination, and presence of genes codifying for virulence factors of Acinetobacter baumannii causing urinary tract infection. Mem Inst Oswaldo Cruz. 2004;99: 839–844.
