# Supplementary material for: Isolation, molecular profiling, and antimicrobial sensitivity patterns of ESBL producing Acinetobacter baumannii in wastewater discharges from Goranchatbari sub-catchment area in Dhaka
Source: PLoS One. 2026 Feb 6;21(2):e0341652. doi: 10.1371/journal.pone.0341652 (PMC12880640; doi:10.1371/journal.pone.0341652)
Supplement: S1 Data — (PDF) [file pone.0341652.s008.pdf]

S1 raw images. The raw representative gel images supporting all blot and gel results reported in the article's figures and supporting information files.

A.

Gel electrophoresis image showing PCR amplicon bands of recA (425 bp) and ITS (208 bp) genes for molecular confirmation of Acinetobacter spp. and A. baumannii, respectively. The isolate IDs and molecular markers (ladder) are labeled in the gel image.
Any lanes not included in the final figure are marked with an "X". A. baumannii NCTC 12156 and no-template control (NTC) were used as positive and negative control, respectively. The gels were visualized on GelDoc Go Imaging System from BioRad. The amplicons were resolved in 1 % agarose gel with 0.5X TBE where Thermo Scientific™ GeneRuler 100 bp Plus DNA Ladder was used.

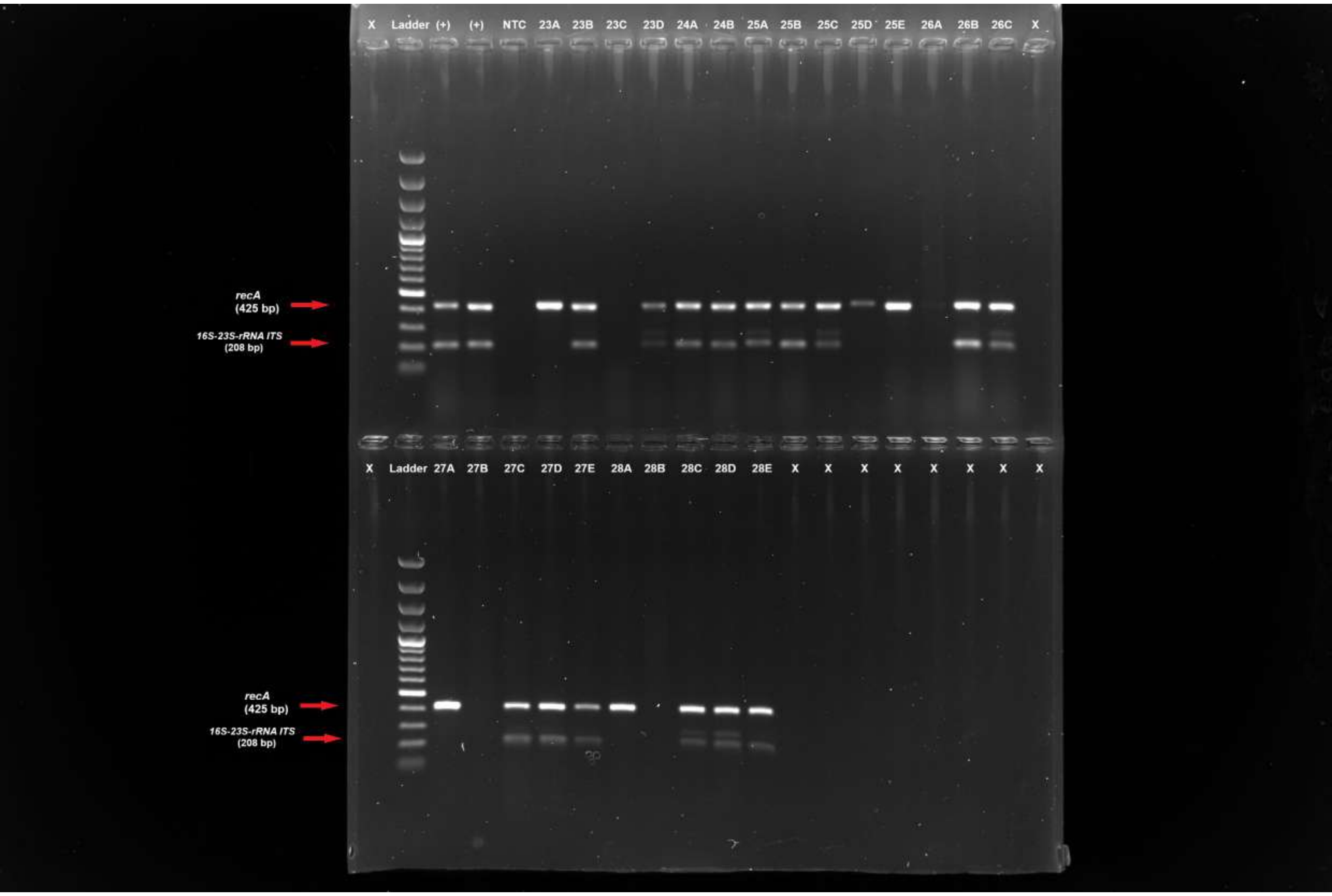

(+)= Positive control
NTC= No template control (negative control)

B.

Raw gel image showing bands for β-lactamase genes- CTX-M, TEM, SHV and OXA. Positive controls for these reactions were sourced from earlier studies. The amplicons were resolved in 1 % agarose gel with 0.5X TBE where Thermo Scientific™ GeneRuler 100 bp Plus DNA Ladder was used.

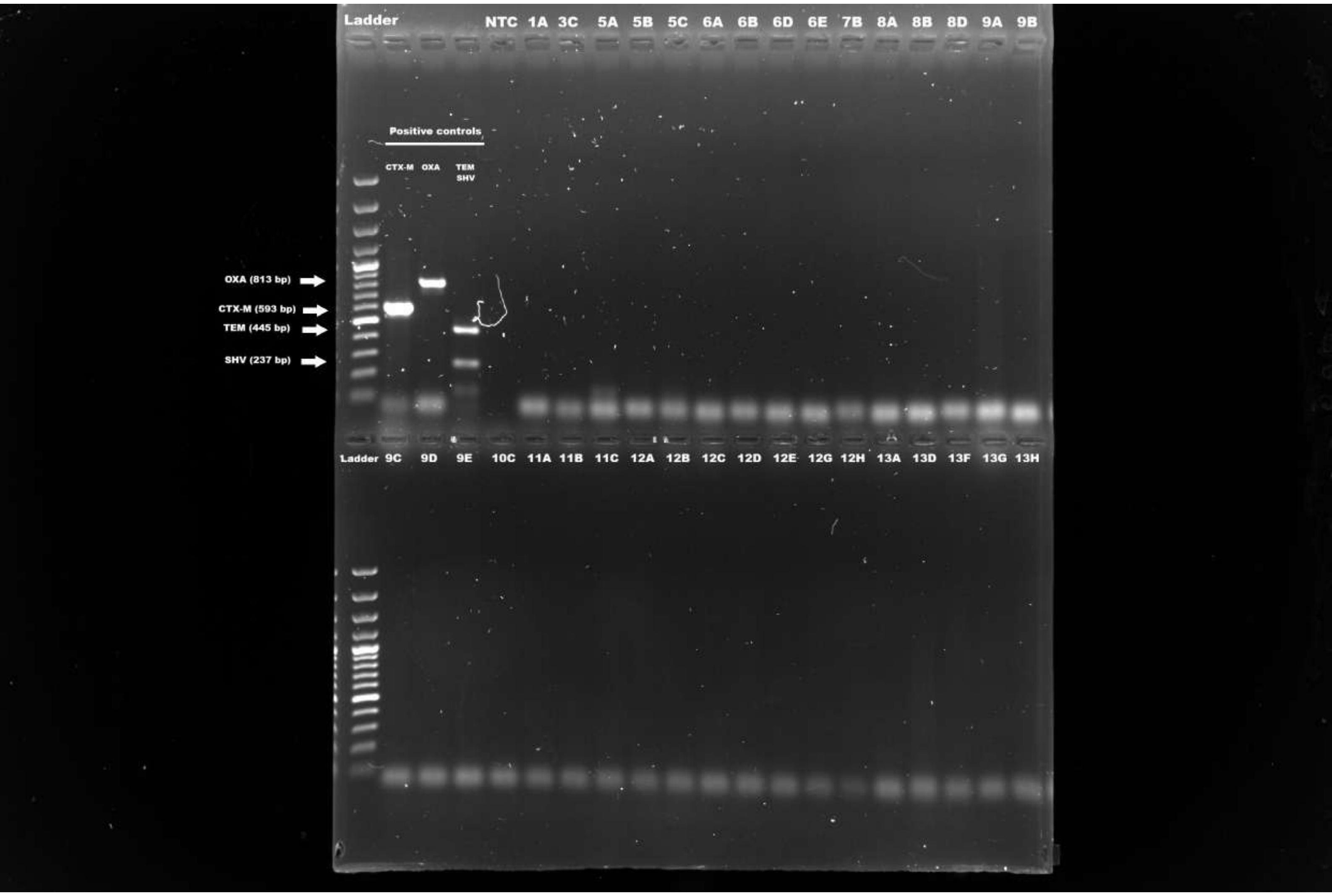

NTC= no template control (negative control)

C.

Gel electrophoresis of bfmS (1428 bp), csuE (976 bp), fimH (870 bp) and espA (451 bp) genes. The isolate IDs and molecular markers (ladder) are labeled in the gel image. The amplicons were resolved in 1 % agarose gel with 0.5X TBE where Thermo Scientific™ GeneRuler 100 bp Plus DNA Ladder was used.

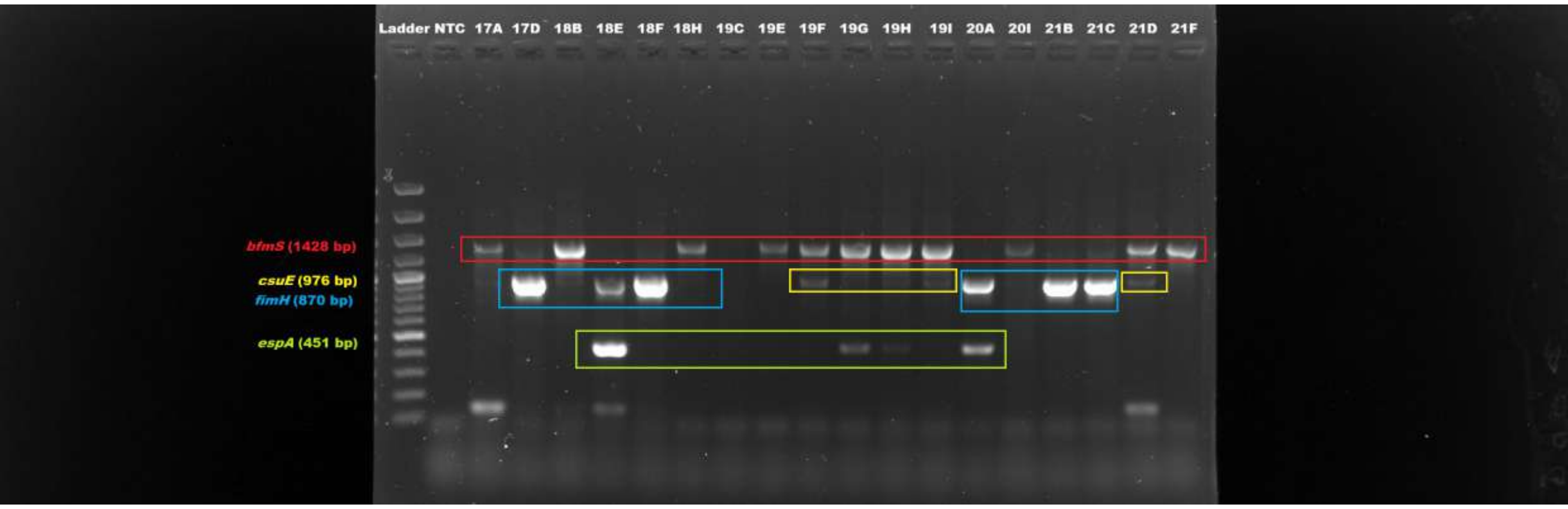

NTC= no template control (negative control)

D.

Gel image showing the bands for ompA (531 bp) and kpsMII (272 bp) genes. The amplicons were resolved in 1 % agarose gel with 0.5X TBE where Thermo Scientific™ GeneRuler 100 bp Plus DNA Ladder was used.

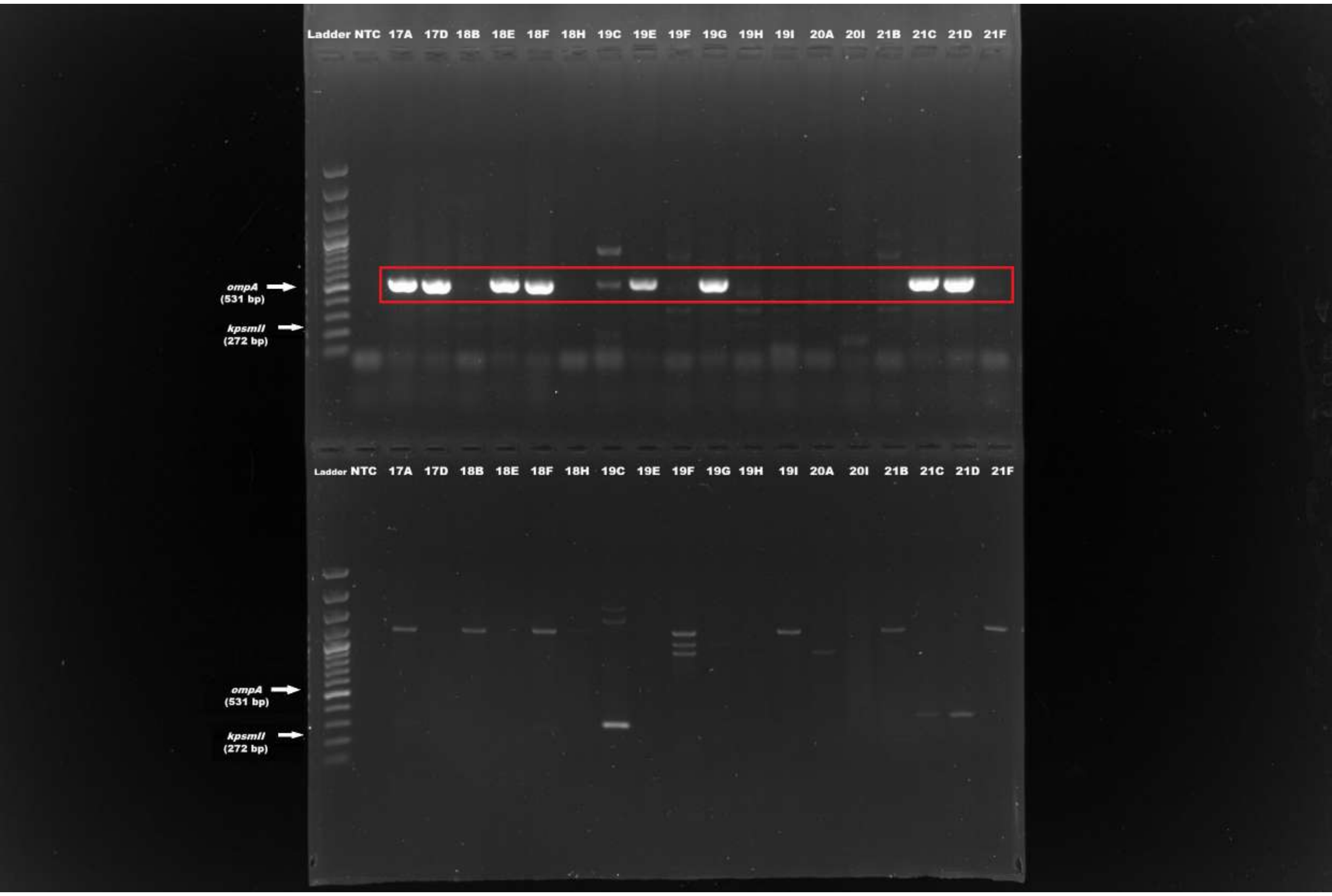

NTC= no template control (negative control)

E.

Raw gel image used in the ERIC PCR dendrogram generation. The isolate IDs and molecular markers (ladder) are labeled in the gel image. Any lanes not included in the final figure are marked with an “X”. The gels were visualized on GelDoc Go Imaging System from BioRad. The amplicons were resolved in 2 % agarose gel with 1X TBE where Thermo Scientific™ GeneRuler 1 kb Plus DNA Ladder was used.

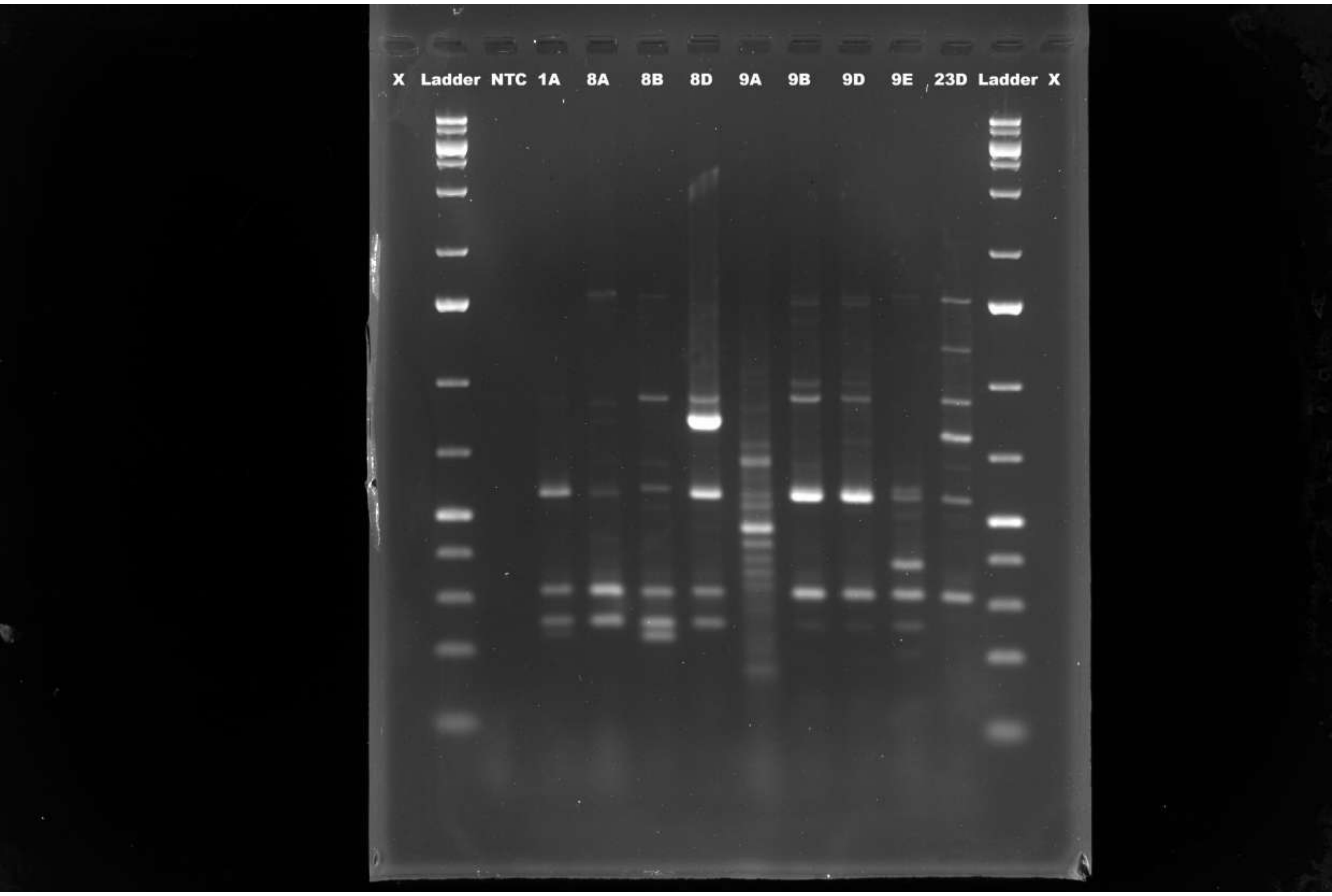

NTC= no template control/negative control.
